# The head morphology of *Pyrrhosoma nymphula* larvae (Odonata: Zygoptera) focusing on functional aspects of the mouthparts

**DOI:** 10.1186/s12983-017-0209-x

**Published:** 2017-05-08

**Authors:** Sebastian Büsse, Thomas Hörnschemeyer, Stanislav N. Gorb

**Affiliations:** 1 Department of Functional Morphology and Biomechanics, Institute of Zoology, Christian-Albrechts-Universität zu Kiel, Am Botanischen Garten 9, 24118 Kiel, Germany; 2 Senckenberg Gesellschaft für Naturforschung, Senckenberganlage 25, 60325 Frankfurt, Germany

**Keywords:** Dragonfly (Anisoptera), Damselfly (Zygoptera), Functional morphology, Ontogenesis, Muscle equipment, Prehensile mask, Feeding apparatus, Micro computed tomography (μCT), Synchrotron radiation micro computed tomography (SRμCT)

## Abstract

**Background:**

The understanding of concerted movements and its underlying biomechanics is often complex and elusive. Functional principles and hypothetical functions of these complex movements can provide a solid basis for biomechanical experiments and modelling. Here a description of the cephalic anatomy of *Pyrrhosoma nymphula* (Zygoptera, Coenagrionidae) focusing on functional aspects of the mouthparts using micro computed tomography (μCT) is presented.

**Results:**

We compared six different instars of the damselfly *P. nymphula* as well as one instar of the dragonfly *Aeshna cyanea* and *Epiophlebia superstes* each*.* In total 42 head muscles were described with only minor differences of the attachment points between the examined species and the absence of antennal muscle M. scapopedicellaris medialis (0an7) in *Epiophlebia* as a probable apomorphy of this group. Furthermore, the ontogenetic differences between the six larval instars are minor; the only considerable finding is the change of M. submentopraementalis (0la8), which is dichotomous in the early instars (I1,I2 and I3) with a second point of origin at the postero-lateral base of the submentum. This dichotomy is not present in any of the older instars studied (I6, middle-late and pen-ultimate).

**Conclusion:**

However, the main focus of the study herein, is to use these detailed morphological descriptions as basis for hypothetic functional models of the odonatan mouthparts. We present blueprint like description of the mouthparts and their musculature, highlighting the caused direction of motion for every single muscle. This data will help to elucidate the complex concerted movements of the mouthparts and will contribute to the understanding of its biomechanics not in Odonata only.

**Electronic supplementary material:**

The online version of this article (doi:10.1186/s12983-017-0209-x) contains supplementary material, which is available to authorized users.

## Background

Insects evolved a staggering diversity of mouthparts and feeding modes [1, 2]. The feeding process usually requires a complex interaction of several specially shaped mouthpart elements – labrum, mandibles, maxillae and labium - moved in a concerted action by muscles through specialised joints supplemented by membranous regions. Such coordinated movements of the mouthparts were studied in exemplary insects using for example tomographic filming techniques [3], but remain poorly understood concerning muscle activation [4] and neuronal control [5].

Odonata larvae shows striking differences in their mouthpart organisation compared to adults and consequently differ in details of their feeding mode. Odonata larvae are aquatic predators, while the adults hunt their prey on the fly [6]. The labium of Odonata larvae shows the most drastic differences compared to that of adults. The larval labium is modified into a prehensile labial mask that is used for capturing potentially fast moving organisms up to predator’s own size. The mandibles and maxillae, however, show only minor differences compared to the adults. Some of these differences in the outer anatomy are reflected in the muscle configuration [7–9].

This transmutation of the labium into a prehensile mask is the most distinctive character of odonatan larvae and rather unique within the insects. Structural aspects of the larval mouthparts of Odonata and the functional mechanism of this catching apparatus have been investigated in various degrees of detail [7, 10–14]. Snodgrass [8], Pritchard [10], and Blanke et al. [9] provided detailed description of the head anatomy, focusing on the cuticular features and the muscle arrangement*.* Furthermore, Olesen [11, 12], Tanaka & Hisada [13] and Parry [14] focussed on the functionality of the labium, studying the mechanism that is responsible for extending the prehensile mask. Their results indicated that the main driving force contributing to the labium extension is an increase in haemolymph pressure generated by the respiratory system of Anisoptera using an internal rectal organ – the branchial chamber. Investigations on this topic in Zygoptera larvae are scarce and their overall morphology differs significantly from that of Anisoptera; in terms of respiration they use external gills – caudal lamellae – for respiration [15] instead of the internal organ mentioned for Anisoptera [6]. The internal anatomy of Zygoptera is controversial and differs from that of Anisoptera: i) extensively like described for example in Whedon [16] or ii) in some functional aspects as described in Miller [17]. However, more recent studies show that Zygoptera larvae are able to intake water into their hindgut and use this mechanism for respiration or supplementing respiration [15, 17–19].

This study was undertaken to better understand the functional morphology of the larval mouthpart system of Odonata. We describe the cephalic anatomy of *Pyrrhosoma nymphula* (Zygoptera, Coenagrionidae) with a focus on detailed 3D description of the mouthpart musculature and its potential function in the feeding process. The idea is to present hypothetical functions of different muscles in the complex movements of the odonatan larval feeding apparatus. This paper provides a solid basis for future biomechanical experiments and modelling. The results, obtained on the representative of Zygoptera, are compared with anisopteran mouthpart musculature and that of *Epiophlebia*, in order to allow more general conclusions for Odonata.

## Results

### Head capsule (Fig. 1a-d)

The strongly sclerotized and dorso-ventrally flattened head is almost three times as broad as long. It is prognathous and only sparsely covered with setae – some on the labrum and laterally at the posterior part of the occiput. The strongly convex and globular eyes protruding laterally are distinctly separated dorsally by almost three times their own width. The occipital ridge marks the anterior border of the occiput and is discernable but weakly developed. The postocciput is triangular and strongly developed. The frons is slightly rounded and declines towards the clypeus. The ocelli are barely discernable in SEM images, but they are present as the tomography data show. The clypeus is divided into a postclypeus and a small anteclypeus. The most important internal structure for muscle attachment is the tentorium. It consists of a rod-like corpotentorium, anterior, dorsal and posterior tentorial arms. The anterior tentorial arms are straight and connected via musculature (0te3) to the head capsule; tentorial pits are not discernable. The weakly developed posterior tentorial arms serve as attachment points of the maxillar muscles 0mx4 and 0mx4.

**Fig. 1:**
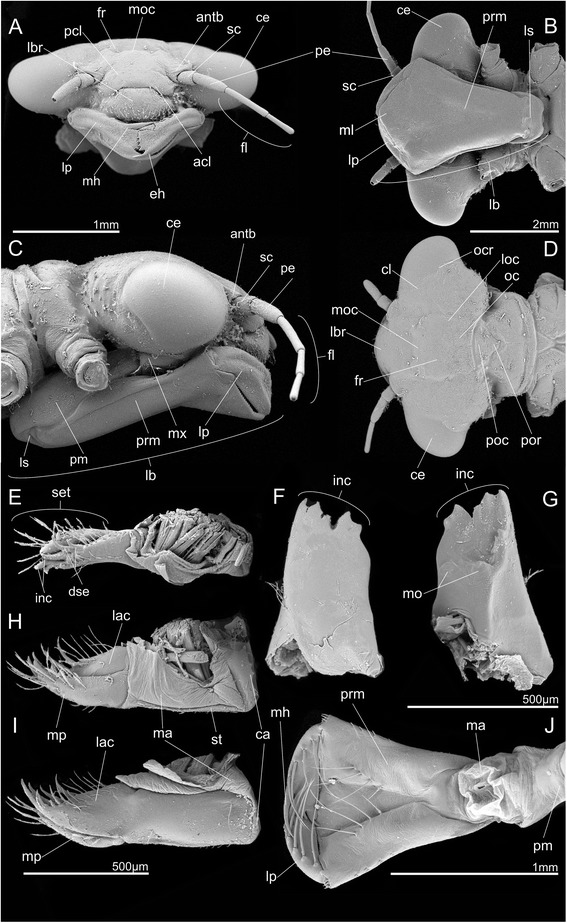
SEM micrographs of *Pyrrhosoma nymphula* larva*.* **a**-**d** Head capsule **e**-**j**. Mouthparts **a**. Frontal view **b**. Ventral view **c**. Lateral view **d**. Dorsal view **e**. Maxilla, dorsal view **f**. Mandible, ventral view **g**. Mandible, dorso-lateral view **h**. Maxilla, median view **i**. Maxilla, lateral view **j**. Labium, dorsal view. Abbreviations: acl – anteclypeus, antb – antennal base, ce – compound eye, ca – cardo, cl – cleavage line, dse – dentisetae, eh – end hook, fl – flagellum, fr – frons, inc – incisivi, lac – lacinia, lb. – labium, lbr – labrum, lp – labial palp, ls – labial sutur, loc – lateral occellus, ma – membranous area, mh – movable hook, ml – median lobe, mo – mola, moc – median occellus, mp – maxillar palpus, oc – occiput, ocr – occipital ridge, pcl – postclypeus, pe – pedicellus, pm – postmentum, poc – post occiput, por – postoccipital region, prm – praementum, sc – scapus, set – setae, st – stipes

#### Musculature

M. tentoriofrontalis dorsalis (0te3) – O: apex of dorsal tentorial arms I: frons, posterior at antennal base.

### Antennae (Fig. 1a,c)

The antennae reach out beyond the mouthparts and are composed of scapus, pedicellus and five flagellomeres. The pedicellus is twice as long as the scapus, and the flagellum is longer than scapus and pedicellus together. The tentorium is the attachment point of the antennal muscles. Antennal heart muscles are absent. Instead, the pharynx and a sac-like structure, situated in front of the brain, hardly discernable in the μCT-data, are connected with the antennal vessels, supporting haemolymph flow [20, 21].

#### Musculature (Figures cf. [8])

*M. tentorioscapalis anterior* (0an1) – O: mesal at the dorsal tentorial arm I: anterior at the base of the scapus. *M. tentorioscapalis posterior* (0an2) – O: mesal at the dorsal tentorial arm, dorsal to 0an1 I: posterior at the base of the scapus. *M. frontopedicellarius* (0an5) – O: dorsal tentorial arm, close to 0an2 I: ventral at the base of the pedicellus. *M. scapopedicellaris lateralis* (0an6) – O: antero-lateral at the base of the scapus I: antero-lateral at the base of the pedicellus. *M. scapopedicellaris medialis* (0an7) – O: meso-lateral at the base of the scapus I: postero-lateral at the base of the pedicellus.

### Labrum (Figs. 1a,d and 2)

The labrum is a roof-like structure at the anterior side of the head arching dorsally above the mandibles. In dorsal view it is semicircular and covered with setae. The tormae (small sclerites), which are almost Y-shaped, are the attachment points for *M. frontoepipharyngalis* (0 lb2).

**Fig. 2:**
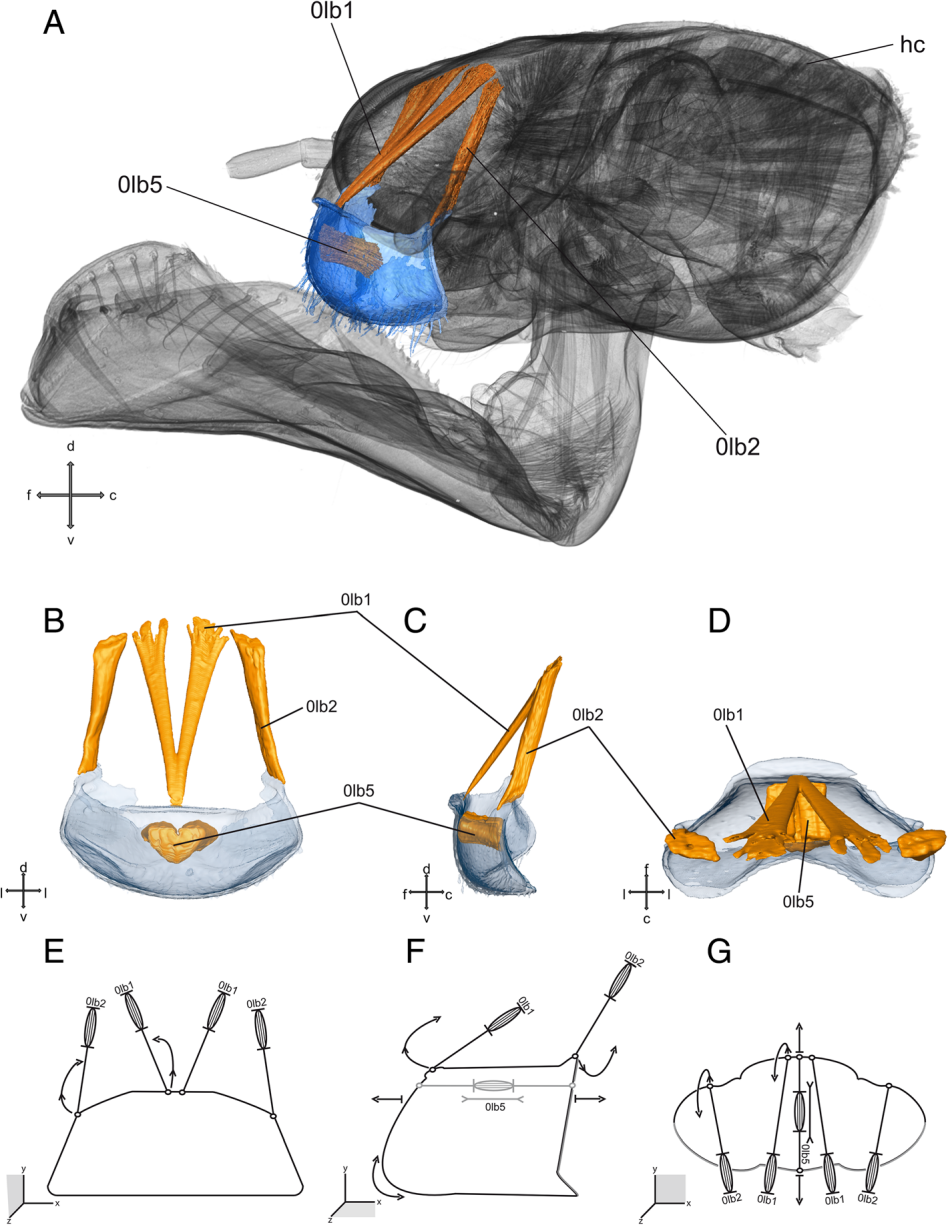
Labrum and head capsule of *Pyrrhosoma nymphula.* **a**-**d** Three-dimensional visualisation from SRμCT data **e**-**g**. Sketch of the hypothetical function of the musculature – grey muscle-pictograms or parts of those indicates internal position within the respective mouthpart, grey additional outlines indicates membranous areas, lengths and broken lines are uninformative **a**. head capsule with labrum, fronto-lateral view **b**, **e**. Frontal view **c**, **f**. Lateral view **d**, **g**. Dorsal view. Abbreviations: c – caudal, d – dorsal, f – frontal, hc – head capsule, l – lateral, lb. - labrum, v – ventral

#### Musculature

*M. frontolabralis* (0 lb1) – O: close to the interantennal ridge I: medial at the base of the labrum C: dichotomous over almost the entire length, unpaired at the very origin. *M. frontoepipharyngalis* (0 lb2) – O: antero-dorsal at the head capsule, lateral of 0 lb1 I: dorso-lateral at the tormae, postero-lateral of 0 lb1. *M. labroepipharyngalis* (0 lb5) – O: anterior at the inner labral wall, ventral of the insertion of 0 lb1 I: posterior at the inner epipharyngeal wall C: unpaired, median within the labrum.

### Mandibles (Figs. 1f,g and 3)

The mandibles are strongly sclerotized and show the typical dicondylic (two articulations points) ball-and-socket type. They are somewhat triangular – slightly elongated – from a dorsal view. The mandibles have four incisivi at the tip, which are broadly connected with each other at their bases, but divided into two groups by an incision. One lateral incisivus is located closer towards the mouth opening. The mola is separated into two areas and composed of one incisivus each. Laterally of the molar lobe, two reinforcing ridges run in cranial direction.

**Fig. 3:**
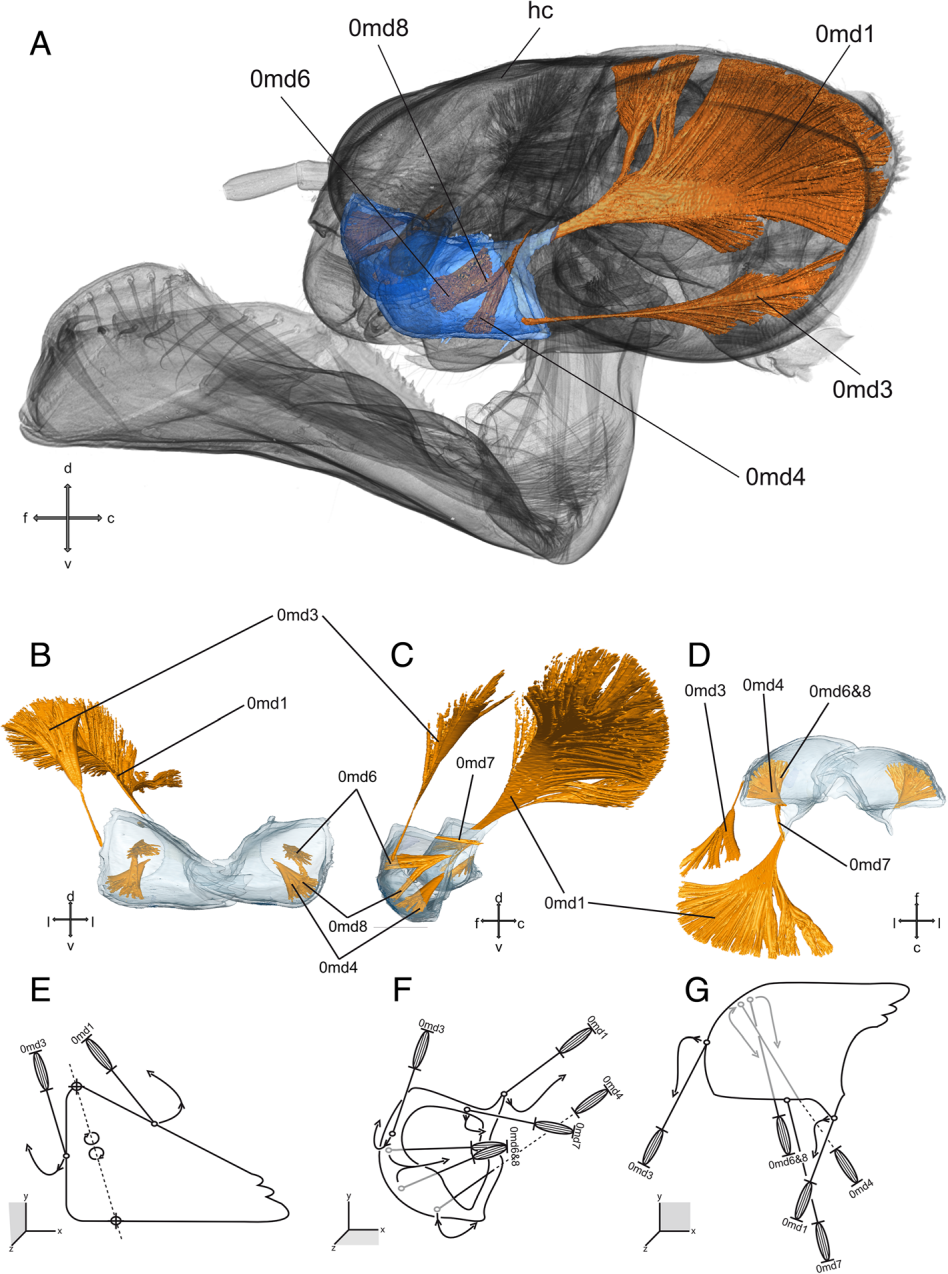
Mandible and head capsule of *Pyrrhosoma nymphula.* **a**-**d** Three-dimensional visualisation from SRμCT data **e**-**g**. Sketch of the hypothetical function of the musculature – grey muscle-pictograms or parts of those indicates internal position within the respective mouthpart, grey additional outlines indicates membranous areas, lengths and broken lines are uninformative **a**. head capsule with labrum, fronto-lateral view **b**, **e**. Frontal view **c**, **f**. Lateral view **d**, **g**. Dorsal view. Abbreviations: c – caudal, d – dorsal, f – frontal, hc – head capsule, l – lateral, md – mandible, v – ventral

#### Musculature

*M. craniomandibularis internus* (0md1) – O: broad areas of the head capsule, postero-dorsal and postero-lateral I: postero-lateral at the mandible, abbuctor tendon C: the largest muscle within the head capsule. *M. craniomandibularis externus posterior* (0md3) – O: postero-lateral at the head capsule I: latero-ventral at the mandible, abductor tendon. *M. hypopharyngomandibularis* (0md4) – O: dorso-lateral at the suspensorial bar of the hypopharynx I: lateral within the mandible. *M. tentoriomandibularis lateralis inferior* (0md6) – O: anterior tentorial arm, via tendon I: lateral within the mandible C: same tendon as 0md8. *M. tentoriomandibularis medialis superior* (0md7) – O: dorsal tentorial arm I: postero-dorsal at the mandible C: weakly developed. *M. tentoriomandibularis medialis inferior* (0md8) – O: anterior tentorial arm, via tendon I: posterio-lateral within the mandible C: same tendon as 0md6.

### Maxillae (Figs. 1e,h & i and 4)

The maxillae are located between the mandibles and the labium, dorso-lateral of the hypopharynx. They are developed to similar extent as in the adult [22]. The maxillae are composed of four parts: cardo, stipes, maxillar palp, and lacinia, a galea is absent. The undivided and triangular cardo is connected via the cardo-stipital membrane to the stipes (no joint is developed) – enabling its movement with respect to the stipes. The latter is almost rectangular in ventral view and divided into basistipes and mediostipes by a longitudinal stipital ridge. At the distal end of the stipes, the maxillary palp originates laterally, and the lacinia originates distally. The maxillary palp is connected via a socket, whereas the lacinia is fused with the stipes.

**Fig. 4:**
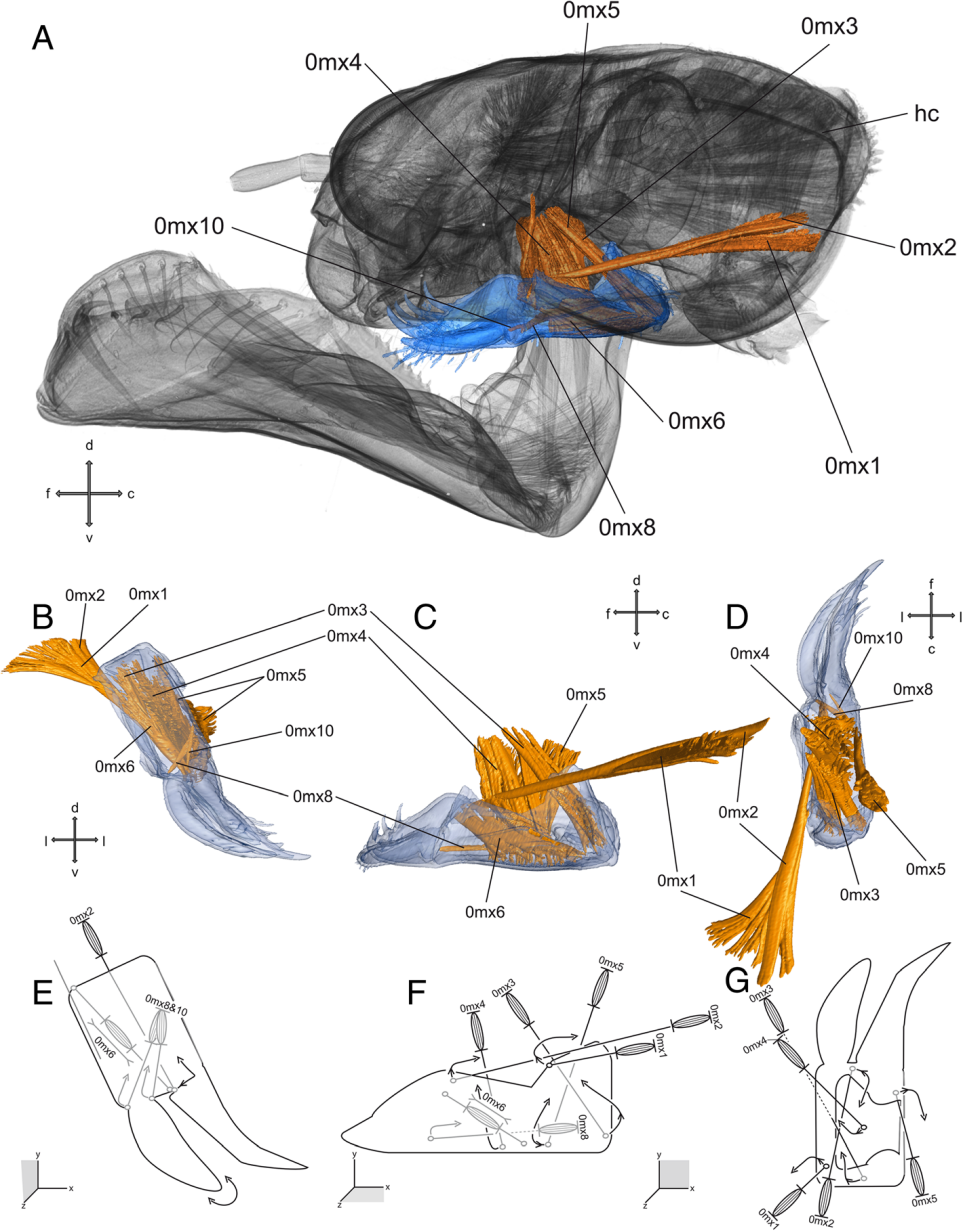
Maxilla and head capsule of *Pyrrhosoma nymphula.* **a**-**d** Three-dimensional visualisation from SRμCT data **e**-**g**. Sketch of the hypothetical function of the musculature – grey muscle-pictograms or parts of those indicates internal position within the respective mouthpart, grey additional outlines indicates membranous areas, lengths and broken lines of are uninformative **a**. head capsule with labrum, fronto-lateral view **b**, **e**. Frontal view **c**, **f**. Lateral view **d**, **g**. Dorsal view. Abbreviations: c – caudal, d – dorsal, f – frontal, hc – head capsule, l – lateral, mx – maxilla, v – ventral

#### Musculature

*M. craniocardinalis* (0mx1) – O: ventro-lateral at the head capsule between 0md1 and 0md3 I: basal via a tendon at the cardo C: fan-shaped muscle (origin). *M. craniolacinialis* (0mx2) – O: at the head capsule, postero-dorsal to 0mx1 I: basal at the lacinia. *M. tentoriocardinalis* (0mx3) – O: lateral at the anterior tentorial arm I: within the cardo. *M. tentoriostipitalis anterior* (0mx4) – O: ventro-lateral at the corpotentorium I: ventral at the stipes, slightly fan-shaped. *M. tentoriostipitalis posterior* (0mx5) – O: ventro-lateral base of anterior tentorial arm, close to 0mx3 I: at the stipes. *M. stipitolacinialis* (0mx6) – O: ventro-lateral at the base of the stipes I: base of lacinia. *M. stipitopalpalis externus* (0mx8) – O: lateral within the stipes close to 0mx10 I: posterior on the base of the palpus. *M. stipitopalpalis internus* (0mx10) – O: lateral within the stipes close to 0mx8 I: anterior on the base of the palpus.

### Labium (Figs. 1b–j and 5)

The labium is developed as prehensile mask responsible for prey capturing. It consists of post- and prementum, ligula, and palps with movable hooks. Glossa and paraglossa are not separated from the prementum by a particular ridge. The post- and prementum are connected via a cubital-like hinge joint, the so-called prementum-postmentum joint (p-p joint), enabling movement of both parts relative to each other. The prehensile mask is connected to the head capsule ventrally via the postmentum. The postmentum shows the largest width at the apical end at the connection with the palps. It narrows towards the joint to almost half of its width. The labial palps are located at the apical tip of the prehensile mask. They are flexibly connected to the mask and show a blunt end hook and a pointed movable hook. Musculature inserting at the end hook is absent.

**Fig. 5:**
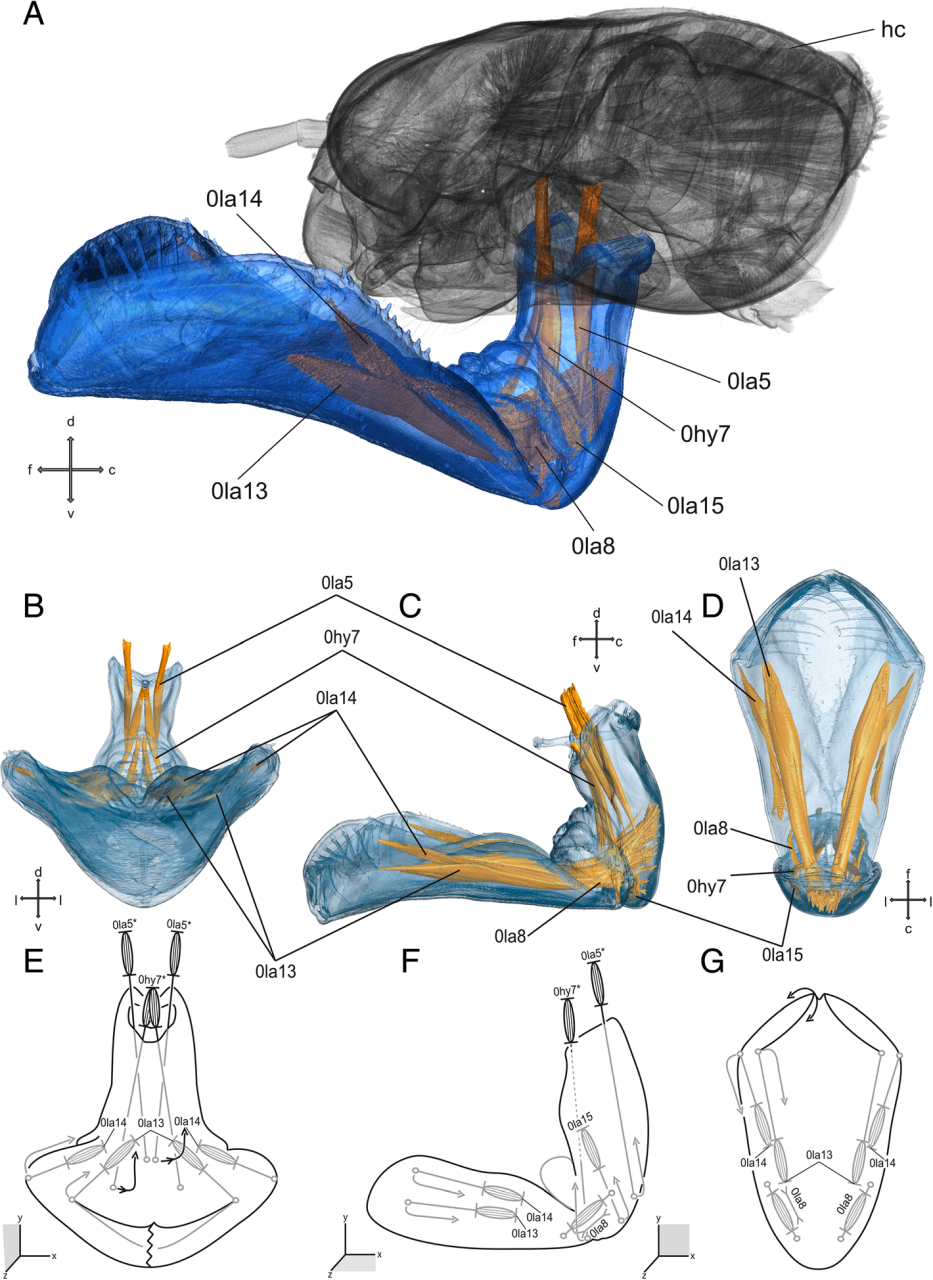
Labium and head capsule of *Pyrrhosoma nymphula.* **a**-**d** Three-dimensional visualisation from SRμCT data E-G. Sketch of the hypothetical function of the musculature – grey muscle-pictograms or parts of those indicates internal position within the respective mouthpart, grey additional outlines indicates membranous areas, lengths and broken lines are uninformative **a**. head capsule with labrum, fronto-lateral view **b**, **e**. Frontal view **c**, **f**. Lateral view **d**, **g**. Dorsal view. Abbreviations: c – caudal, d – dorsal, f – frontal, hc – head capsule, hy – hypopharynx, l – lateral, la – labium, v – ventral

#### Musculature

*M. tentoriopraementalis* (0la5) – O: posterior at the corpotentorium I: lateral at the premental edge. *M. submentopraementalis* (0la8) – O: at the posterior part of the submentum I: dorsal at the prementum C: dichotomous in the early instars (I1,I2,I3) O2: at the postero-lateral base of the submentum. *M. praementopalpalis internus* (0la13) – O: median at the prementum, ventral of 0la14 I: antero-median at the base of the palpus. *M. praementopalpalis externus* (0la14) – O: median at the prementum, dorsal of 0la13 I: lateral at the base of the palpus. *M. praementomembranus* (0la15) – O: anterio-lateral at the postmentum I: postero-lateral at the prementum.

### Hypopharynx (Fig. 6)

The hypopharynx is located anteriorly of the labium. It is rounded at the side facing towards the prementum. At its posterior base, a T-shaped rod originates and serves as attachment point for 0hy7.

**Fig. 6:**
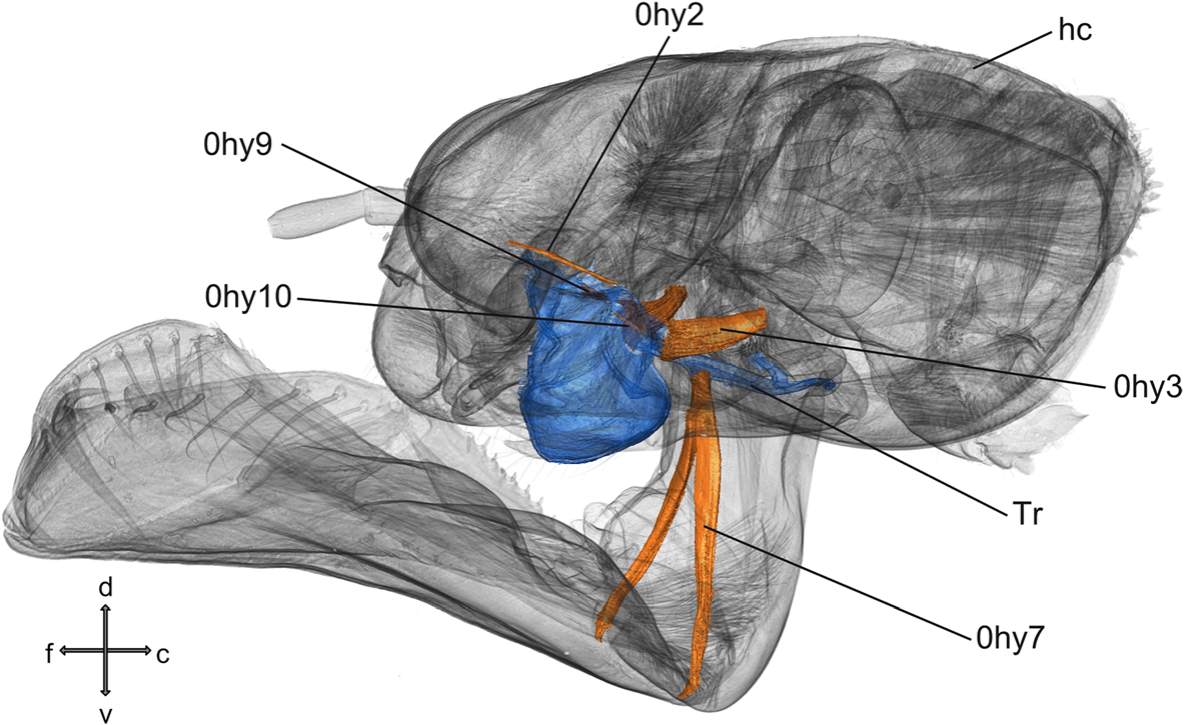
Hypopharynx and head capsule of *Pyrrhosoma nymphula.* Three-dimensional visualisation from SRμCT data in fronto-lateral view. Abreviation: c – caudal, d – dorsal, f – frontal, l – lateral, v – ventral, Tr – T-rod

#### Musculature

*M. frontobuccalis lateralis* (0hy2) – O: at the frons, ventral of the base of the antennae I: lateral at the suspensorial sclerites. *M. tentoriohypopharyngealis* (0hy3) – O: latero-ventral at the corpotentorium I: at the hypopharynx. *M. praementosalivaris anterior* (0hy7) – O: antero-lateral at the prementum; I: ventral at the T-rod. *M. oralis transversalis* (0hy9) – O: at the suspensorial sclerite, oral, dexter I: at the suspensorial sclerite, oral, sinister. *M. loroloralis* (0hy10) – O: at the suspensorial sclerite, loral, dexter I: at the suspensorial sclerite, loral, sinister.

### Pharynx and oesophagus

The wide lumen of the pharynx and oesophagus is folded dorsally, laterally and ventrally; these folds serve for muscle attachment and for an increase in the surface area. There is no contact with the corpotentorium. The musculature of the pharynx and oesophagus is strongly developed.

#### Musculature (Figures cf. [8])

*M. clypeobuccalis* (0bu1) – O: at the clypeus I: at the bucca. *M. frontobuccalis anterior* (0bu2) – O: close at the interantenal ridge, posterior to 0 lb1 I: dorsal at the bucca. *M. frontobuccalis posterior* (0bu3) – O: posterior at the frons I: dorsal at the bucca. M. tentoriobuccalis lateralis (0bu4): O: base of dorsal tentorial arms; I: lateral at the bucca. *M. tentoriobuccalis anterior* (0bu5) – O: at the corpotentorium I: ventral at the bucca. *M. tentoriobuccalis posterior* (0bu6) – O: dorsal at the anterior tentorial arm I: ventral at the bucca. *M. verticopharyngalis* (0 ph 1) – O: at the occiput, medial of 0md1 I: dorsal at the pharynx. *M. tentoriopharyngalis* (0 ph 2) – O: at the corpotentorium I: ventral at the pharynx.

## Discussion

### Comparative morphology

Ninety-one head muscles are known so far for insects [23–25]. The most recent account for larval Odonata described 41 muscles for *Epiophlebia* [9]. Here we describe 42 head muscles for larval *Pyrrhosoma nymphula*.

The difference is due to the absence of antennal muscle 0an7 in *Epiophlebia* (*E. superstes*), which seems to be a peculiarity of *Epiophlebia*, since it is present in Anisoptera (*Aeshna cyanea*) as well as Zygoptera (*Pyrrhosoma nymphula)* – see also Blanke et al. [9]. Furthermore, there is a shift of the origin point of the muscle 0an5 from the frons to the tentorium – 0an5 might take over the function of 0an7 – we follow the suggested homologization from Blanke et al. [9].

The peculiarities of the head musculature in Odonata larvae in comparison to adults mentioned by Blanke et al. [9] can be confirmed. More precisely, the absence of M. tentoriomandibularis lateralis superior (0md5), M. postoccipitopharyngealis (0 ph 3) and M. postoccipitalohypopharyngealis (0hy4) as well as the presence of the labial muscle M. tentoriopraementalis (0la5), antennal muscle M. tentoriofrontalis dorsalis (0te3), and the presence of the hypopharyngeal muscles (0hy2, 0hy3 0hy9, 0hy10), buccal muscles (0bu3, 0bu4, 0bu5), and pharyngeal muscule (0 ph 2) can be confirmed.

Muscle 0 lb1 of the labrum is a dichotomous muscle in *P. nymphula* while it is an unpaired muscle in *Epiophlebia*. The mandible muscle 0md7 is weakly developed compared to that in representatives of Epiprocta.

### Muscle ontogenesis

Different instars of *P. nymphula* only show minor anatomical differences. Accompanied by the continuous increase in size, a slight shift of head proportions is noticeable during ontogenesis. Additionally, the prementum becomes elongated, if compared to the submentum, and the labium becomes elongated rather than widened. Small teeth-like sclerotized structures appear anteriorly at the lateral edge of the prementum [26] of instar 6. Generally, characters used in taxonomy [26], like the rows of setae on the head capsule especially at the dorso-caudal postocciput or on the labial palpus and ventro-medial of the prementum, are fully developed in later instars. Furthermore, the mode of crypsis changes towards instars 5 and 6 from a hyaline almost transparent body, which supposedly leads to near-invisibility in the body of water, to a piebald cuticle comprising different shades of beige and brown with spots of whitish and blackish to camouflage in the benthos [6].

The only muscular change within the larval stages is revealed concerning M. submentopraementalis (0la8), which is dichotomous in the early instars (I1,I2 and I3) with a second point of origin at the postero-lateral base of the submentum. This dichotomy is not present in any of the older instars studied (I6, middle-late and pen-ultimate).

Compared to the adult, drastic differences in the overall morphology and muscle arrangement (cf. ‘comparative morphology’ section) occur not only because of the transmutation of the labium (from its insect ground pattern) into a prehensile mask. The re-orientation of the mouthparts from prognathous in the larva to orthognathous in the adult takes place. This modification leads to the adaptation to the different prey-capturing mode in the adult. The adults use their specialised legs as a basket for catching prey in flight [27] – the prey is brought to the mouth from ventral. Whereas, the larva uses its prehensile mask for prey-capturing under water – the prey is brought to the mouth from frontal. Furthermore, the presence of the hypopharyngeal muscles (0hy2, 0hy3 0hy9, 0hy10), buccal muscles (0bu3, 0bu4, 0bu5), and pharyngeal muscle (0 ph 2) and the strong development, within the larvae, of the latter two muscle groups might indicate further adaptations for ingestion within an aquatic habitat – ingestion via sucking the food “solved” in water. However, the most extensive change is the transmutation of the labium. Here, an elongation of the pre- and postmentum as well as the transformation of the labial palps into prey grasping organs occur. The abductor – M. praementopalpalis externus (0la14) – and adductor – M. praementopalpalis internus (0la13) – of the former palpi are greatly enlarged and the points of origin is translocated to the base of the postmentum to increase the applicable force. Two well-developed joints, prementum-head joint (P-H joint) ventral of the hypopharynx and the prementum-postmentum joint (P-P joint), lead to an increase of movability of the prehensile mask. Furthermore, the T-shaped rod or T-rod is characteristic for larval Odonata [8] (more precisely the hypopharyngeal apodem) serves as an important attachment structure for the redirected muscles (see also subsection ‘labium’ within the next section).

### Functional significance of different groups of mouthparts muscles

The labrum (Fig. 2) limits the preoral cavity anteriorly. It is connected to the clypeal area by the membranous clypeolabral suture, which enables movement of the labrum. This movement is realised by the antagonistic action of muscles 0 lb1, 0 lb2. The muscle 0 lb5 compresses the labrum due to its attachment at its anterior and posterior (epipharyngeal) wall, the cuticle elasticity might function as muscle antagonist for returning of the labrum shape to its original condition. The muscle 0 lb1 is attached anterio-medially of the labrum and enables its dorso-lateral movement. The muscle 0 lb2 is the antagonist that is attached postero-laterally of the labrum and enables its dorso-medial movement.

The mandibles (Fig. 3) are strongly sclerotized jaws for crushing harder parts of the prey [6]. The movability of the mandibles is restricted to one axis due to the anterior and posterior ball-and-socket joints. This holds true for Odonata similar to other groups of insects [28]. These joints span a virtual axis of rotation and this rotation is produced by the antagonistic action of 0md1, the adductor muscle, and 0md3, the abductor muscle. Muscles 0md4, 6, 7 and 8 are often reduced in other winged insects [1]. Muscle 0md4 is attached antero-lateral within the mandible and at the hypopharynx, where it might support the movement of the hypopharyngeal sclerites. Muscles 0md6 and 0md8 are attached lateral and postero-lateral within the mandible, respectively. They enable a dorso-lateral and postero-dorsal movement of the mandible and support in this function the main abductor 0md3 [4].

The maxillae (Fig. 4) are specialised for manipulating, handling and sensing the food. The feeding movements of the maxillae are mainly protraction and retraction [8]. The muscles are able to move the maxillae in almost every direction, because flexible membranous regions and the cardo-cranial joint implement the suspension. The muscles 0mx1, 2, 3, 4 and 5 are used to move the maxilla very precisely: 0mx1 – is attached postero-lateral on the maxilla at the cardo (lateral to the head) and enables a dorso-median movement of the cranial part of the maxilla; 0mx2 – is attached antero-median on the maxilla at the lacinia and enables a dorsal (slightly dorso-lateral) movement of the apical part of the maxilla; 0mx3 – is attached postero-median on the maxilla within the cardo and enables a dorsal (slightly dorso-lateral) movement of the cranial part of the maxilla; 0mx4 – is attached median on the maxilla at the stipes and enables a dorsal movement of the maxilla; 0mx5 – is attached antero-lateral (medial to the head) and enables a dorso-lateral movement of the maxilla. During the protracting process the laciniae are used to grasp the prey, provided by the prehensile mask, by thrusting forward beyond the mandibles [8]. After retracting the maxillae, the food grasped by the laciniae, is delivered to the mandibles, where the digestive process starts. The muscle 0mx6 runs completely within the maxillae and is attached at the median base of the lacinia and might enable abduction. During or before this process the palps are used to sense the food. The maxillary palp is, due to its connection with the stipes via a ball and socket joint, very movable. The abductor muscle 0mx8 enables a ventro-lateral movement of the palp, whereas the opposing adductor muscle 0mx10 enables a dorso-medial movement of the palp. Combined with the dorso-ventral movement of both 0mx1 and 0mx2 the sensing in every direction is enabled.

The locking mechanism of the prehensile mask, as described by Olesen [12], where the maxillae are supposed to be used to prevent propelling of the prehensile mask could not be confirmed.

The labium (Fig. 5) performs the most interesting biomechanical movement in Odonata larvae (cf. Additional file 1: High-Speed Video). It represents a strong modification of mouthparts within the insects: the modification of the labium into a prehensile mask used for prey capturing. The process of prey capturing can be divided in to two different events: i) propelling of the prehensile mask towards the prey, and ii) grasping the prey using the movable pointed hooks.


Additional file 1: High-speed video of the prey capturing process of a Zygoptera larva. (MOV 12373 kb)


The muscles 0la5, 0la8, 0la15, and 0hy7 are used for retracting the prehensile mask after the strike and to hold it in its resting position. They could presumably work as an antagonist against the pressure, while the jet propulsion in Anisoptera takes place, as suggested by Pritchard [10]. While muscles 0la5, 0la8 and 0hy7 might help to prevent propelling the prementum, muscle 0la15 might prevent the shearing of the postmentum against the head capsule. The movability of the prehensile mask is restricted by two joints, the prementum-head joint (P-H joint) and the prementum-postmentum joint (P-P joint). More precisely: 0la5 – is attached medially at the base of the postmentum and enables a dorsal movement towards the head, slightly restricted by the P-H joint for retraction/holding; 0la8 – is running from the ventral base of the prementum to the apical side of the postmentum; this muscle locks the movability within the p-p joint; 0la15 – is attached at the very base of the postmentum and might prevent the shearing of the postmentum against the head capsule and/or helps to lock the post- and prementum to each other; 0hy7 – is attached at the ventral base of the prementum directly at the labial articulation and enables a dorsal movement towards the head, strongly restricted by the P-H joint for retraction/holding. All these muscles might be included in the propelling movement of the prehensile mask, to steer during and/or positioning the mask. However, it becomes clear that the propelling of the prehensile labial mask cannot be powered by normal muscle contraction, since muscle contraction enables only the opposite movement.

However, both muscles 0la5 and 0hy7 become deflected in the completely drawn-in position of the prehensile labial mask. The muscle 0la5 is bent by the T-rod apodem (see above paragraph ‘muscle ontogenesis), as described by Blanke and colleagues [9]: the bent ends of this apodem deflect the muscle 0la5 towards the thorax. The same is true for the muscle 0hy7: here the dorsal base of the prementum functions as ‘deflection sheave’ and redirects the muscle towards the tip of prementum.

So far the results of previous investigations indicate that the main force for the labium extension is produced by abdominal dorso-ventral muscles and transmitted to the labium as haemolymph pressure increases [11–14]. The same abdominal muscles are also involved in the mechanism of water intake into the digestive tract, to increase the body pressure, for respiration and swimming by jet propulsion [12, 29–31]; the latter is an escape mechanism in anisopteran larvae [6]. Epiprocta [Anisoptera + *Epiophebia*] use a dedicated internal respiration organ and are able to escape via jet propulsion (never observed in *Epiophlebia*) [32]. The ability to increase haemolymph pressure by closing their anal valve, in order to propel the prehensile mask, is therefore studied in anisopteran taxa only. The mechanism of propelling the prehensile mask in Zygoptera is not properly studied so far, due to missing experimental investigations and a differing larval anatomy as mentioned above. Caillère [33, 34] investigated the prey capturing strike of Zygoptera mentioning a convergent movement of the gills and the beginning of a forward movement of the digestive tract (abdomen) just before the prey capturing process. The extension of the prehensile mask and the forward movement of the abdomen stop simultaneously, and at almost the same time, the convergent movement of the gills ceases [33, 34]. This observation as well as the investigations by Eriksen [15], Miller [17, 18] and Sesterhenn and colleagues [19] confirm that also Zygoptera are able of an water intake into their digestive tract even though their external gills are responsible for a significant part of the gas exchange [15], as already Tillyard [7] suggested. However, the internal anatomy differs at least in the “lack of the diaphragm and the sub-intestinal muscle, which allows anisoptera larvae to suck water directly into the branchial chamber” ([17],p.386). Zygoptera are therefore restricted to a more gulping like ventilation, most likely due to the mentioned differences and a smaller volume of the related respiratory organs [17].

Since it is implausible – parsimony principle – that Zygoptera and Anisoptera use different mechanisms for such a highly complex biomechanical process, we safely can assume that the extension of the labium in Anisoptera as well as in Zygoptera is based on the same principles. Nevertheless, the mechanism of the mask propelling within the Odonata – considering Zygoptera and Anisoptera – should be reinvestigated because of the significant differences mentioned.

The second important process in prey capturing, is the prey grasping, which is realised by the strongest musculature found in the prehensile mask (Fig. 5). The adductor muscle 0la13 realizes the grasping and clutching of the prey at the tip of the labium by closing the labial palps with the movable hooks. The abductor muscle 0la14 realizes the opening of the labial palps, to release the prey remains and bring the palps in the initial position for new prey capturing process.

The hypopharynx (Fig. 5) is situated in the preoral cavity in front of the functional mouth [1]. The strongest muscle 0hy3 is the retractor of the hypopharynx, originating on the tentorium to allow a caudal movement and therefore a widening of the oral cavity. Muscle 0hy2 originating at the frons, most likely is able to protract the hypopharynx into the oral cavity, serving as antagonist to 0hy3. In Odonata, the unique T-rod or T-shaped rod is originating from the hypopharynx and 0hy7, which originates on the T-rod and inserts within the prehensile labial mask plays an important role in its movement (cf. paragraph on the labium). The small muscles 0hy9 and 0hy10 attaching at the left and the right side of the suspensorial sclerites might deform the hypopharynx laterally.

## Methods

We studied six (L1, L2, L3, L6, middle-late, pen-ultimate) instars of *Pyrrhosoma nymphula* (Sulzer, 1776) (Zygoptera; Coenagrionidae), for comparison we also investigated larval specimens (late instars) of *Aeshna cyanea* (Müller, 1764) (Anisoptera; Aeshnidae), and *Epiophlebia superstes* (Sélys, 1889) (Epiprocta; Epiophlebiidae). All figures show the pen-ultimate instar. The specimens were fixed in alcoholic Bouin solution (= Duboscq-Brasil) and stored in 70–80% ethanol [35]. All applicable regulations concerning the protection of free-living species were followed. All necessary permits were obtained for collecting Odonata at the Billingshäuser Schlucht, Göttingen, Germany (permission granted by “Untere Naturschutzbehörde” file reference AZ.67.2.5 Wei). Prior to scanning (both CT and SEM), the samples were dehydrated in an ascending ethanol series and dried at the critical point (Balzers CPD030) or using Hexamethyldisilazan (HMDS) [35].

High resolution X-ray tomography (μCT) was carried out using a SkyScan 1172 desktop micro-CTscanner (Bruker micro-CT, Kontich, Belgium) at 40 kV and 250 μA with images taken every 0.25°. Additionally, we used the synchrotron radiation micro-computed tomography (SRμCT) setup at the Tomcat beamline of the Swiss Light Source, Villigen, Switzerland [36].

Segmentation and visualization of the data were done with Amira 5.4.3 (FEI SAS, France, www.vsg3d.com) and Photoshop CS3 (Adobe SystemInc.). Please refer to Betz et al. [37] for further information on the general setup for SRμCT and to Büsse et al. [38] for information on segmentation, labelling and visualization with Amira.

For SEM, the samples were dehydrated in an ascending ethanol series, critical point dried (Quorum E3000) and sputter-coated with gold-palladium (10 nm thickness; Leica Bal-TEC SCD500). Afterwards the samples were mounted on a rotatable sample holder [39] and examined in a Hitachi TM3000 scanning electron microscope at an accelerating voltage of 15 kV.

For the high-speed video recordings a Photron Fastcam SA1.1 (model 675 K–M1, www.vkt.de) equiped with a 105 mm/1:2.8 macro lens (Sigma, Japan, www.sigma-photo.co.jp) mounted on a Manfrotto055 tripod with Manfrotto410 geared head (Manfrotto, Italy, www.manfrotto.com) and two Dedocol COOLT3 light sources (Dedotech, Switzerland, https://dedotec.ch) was used. The labial strike was shot with 5400 frames per second (fps) (1/frame, Trigger Mode: End, Resolution 1024 × 1024). The video was saved as 16-bit TIFF image-stack and later reconstructed into a video format (AVI) using ImageJ 1.51e (National Institutes of Health, USA, https://imagej.nih.gov/ij/).

The juvenile stage in Odonata, Ephermeroptera and Plecoptera is suggested to be called “naiad “following the terminology proposed in Bybee et al. [40]. However, since for Odonata the more commonly used name is “larva”, we decided to be consistent within the terminology, mainly used in the community, and used here the more general term [41, 42]. Anatomical structures are described using the nomenclature of Beutel et al. [43], muscle designations are made using the nomenclature of Wipfler et al. [25]. Muscles are described stating their origin (O) and their insertion (I) followed by (C) some special characteristics, if present.
